# Differential time-course tear film quantitative changes following limbal relaxing incisions

**DOI:** 10.1186/s12886-020-01447-4

**Published:** 2020-05-03

**Authors:** Mohamed Attia Ali Ahmed, Ahmed Shawkat Abdelhalim

**Affiliations:** Faculty of Medicine, Ophthalmology Department, Minia University, Minia, Egypt

**Keywords:** Corneal astigmatism, Limbal relaxing incisions (LRIs), Basic Schirmer’s test (BST), Tear break-up time test (TBUT)

## Abstract

**Background:**

The study aims at evaluating the time-course changes of pre-corneal tear film after simultaneous phacoemulsification and limbal relaxing incisions (LRIs) performed in 2 groups of patients; group-A had vertical and group-B had horizontal LRIs.

**Methods:**

Fourty-two eyes of 28 patients with co-existing cataract and corneal astigmatism were studied before and after simultaneous cataract surgery and LRIs (at weeks 1, 4 and 12), patients were classified into 2 groups according to the orientation of LRIs; vertical (A) and horizontal (B) groups. Pre-corneal tear film stability was assessed by measuring the tear break-up time (TBUT) and the tear volume was determined using Schirmer’s I test (Basic Schirmer’s test; BST), both preoperatively and postoperatively.

**Results:**

TBUT was significantly reduced in both the study groups (*P* = 0.001) without significant reduction regarding basic Schirmer’s test values except for the first postoperative week in the horizontal LRI group-B (*P* = 0.04).

**Conclusions:**

Precorneal tear film stability is altered in the early postoperative period after simultaneous cataract and LRI incisions shown by TBUT measurement values. These changes do not appear to differ significantly depending on the orientation of LRI incisions.

## Background

Microscopic damage to the ocular surface during cataract surgery is a widely established theory of postoperative dry eye syndrome resulting in ocular discomfort and dissatisfaction [1].

Corneal astigmatism of variable degree; ranging between 1and 3 diopters; has been found to be coexistent in up to 29% of patients who are complaining of lenticular opacities and probably would undergo cataract surgery [2–4].

Phacoemulsification; the modern cataract surgery; with intraocular lens implantation has been considered the gold standard treatment of cataract. The standard cataract procedure was simply targeting to correct spherical equivalent refractive error without considering a coexisting corneal astigmatism. Cataract surgery could be an aggravating factor of a pre-existing corneal astigmatism for the incisional nature of the procedure or a precipitating factor for a de novo surgical-induced astigmatism of a variable degree. Postoperative patient’s satisfaction after cataract surgery is basically related to achieving optimal postoperative distant visual acuity without the need to be spectacle or being contact lens-dependent. Despite recent improvements in surgical techniques, biometric measuring tools, and IOL power calculation formula; uncorrected corneal astigmatism still could be a source of postoperative residual refractive error [5–8]. Patients are usually reluctant to use spectacles or contact lenses for correcting postoperative astigmatism; having their own limitations and complications [3]. With eminence of the modern refractive cataract surgery techniques; limbal relaxing incisions, astigmatic keratotomy, bioptics and toric IOL implantation; both spherical and astigmatic refractive errors were targeted to be corrected simultaneously [2, 9, 10].Being incisional procedures; both phacoemulsification and limbal relaxing incisions, could precipitate postoperative ocular surface changes in the form of decreased tear production, appearance of dry eye and ocular discomfort syndromes; all of which might interfere with the patient’s daily life quality. We have earlier studied the ocular surface changes following simultaneous cataract surgery and LRIs; the current study investigated differential changes that depend on the orientation at which the LRIs were performed [11]. It has been previously reported in literature that corneal nerves enter the cornea predominantly at the horizontal meridian (3 and 9 o’clock positions) [12, 13]; however; other authors reported that nerve fiber bundles in the sub-basal plexus across the central and mid-peripheral cornea run first in the horizontal direction, then after bifurcation, they do travel in the vertical (6 and 12 o’clock hours) direction and after a second bifurcation again they run in the horizontal direction [14]. according to that we postulated that there may be differential changes after performing LRIs in different orientations regarding the ocular surface quality profile. The aim of this study is to longitudinally assess the pre-corneal tear film changes in eyes undergoing simultaneous phacoemulsification and LRI procedures and to investigate whether these changes may vary according to the orientation of LRIs.

## Methods

### Study design

Prospective observational case series.

### Subjects

Forty-two eyes of 28 patients were prospectively examined; patients were further classified according to the orientation of the LRIs into 2 groups; group-A who had LRIs performed along the vertical meridian (vertical LRIs) and Group-B who had the LRIs performed along the horizontal meridian (horizontal LRIs). Inclusion criteria for the current study were: patients of both genders palnned to undergo cataract surgery with preoperatively documented mild to moderate corneal astigmatism (0.50–1.75 D) excluding those with severe corneal astigmatism (> 1.75 D) whom planned to have toric IOL implantation for correcting the pre-existing corneal astigmatism or not preferring to have the LRI procedure, patients with evident dry eye syndrome, severe ocular surface disorders, or systemic disease compromising the quality of the ocular surface (Steven-Johnson syndrome, systemic lupus erythematosus), autoimmune disorders or corneal degenerative conditions associated with peripheral corneal thinning such as rheumatoid arthritis and pellucid marginal degeneration that render LRI to be unsafe procedure with unpredicted outcomes.

Table 1 shows the study population groups’ demographic data.

**Table 1:** Preoperative demographic data of the study population groups; Group-A: vertical LRIs and Group-B: horizontal LRIs

Parameter	Group-A	Group-B	***P***-value
**Number of patients**	13	15	–
**Number of eyes**	21	21	–
**Gender (Female)**	7	7	0.97
**Age (Years)**	70.65 ± 9.50	75.25 ± 8.51	0.15
**Preoperative TBUT (Sec)**	7.41 ± 2.48	8.81 ± 5.59	0.73
**Preoperative BST (mm)**	14.71 ± 8.86	16.13 ± 9.32	0.61

*TBUT* Tear Break-up time test, *Sec* second, *mm* millimeter, *BST* Basic Schirmer’s test

The study protocol was approved by the institutional review board of Minia Faculty of Medicine Research Ethics Committee (FMREC) and compiled with the tenets of the Declaration of Helsinki. All study participants signed a written informed consent to participate in the study and for publication of data before being enrolled in the study; after explaining the nature and details of the study procedures.

All participants had the standard cataract surgery (phacoemulsification) performed simultaneously with LRIs. The horizontal axis (0–180°) was marked at the slit-lamp preoperatively while the patient was in sitting position to compensate for potential cyclotorsion when shifting to the supine position. The incisions were performed in the steepest corneal axis at the limbus just anterior to the palisades of Vogt for correcting preoperative corneal astigmatism which was documented preoperatively by the corneal topographer (ATLAS-9000, Carl Zeiss Meditec, Germany). LRIs were performed according to the modified Gills’ nomogram at the commencement of surgery using a guarded micrometer diamond blade set at 500 μm as paired arcuate incisions. At the end; the incisions were irrigated with a balanced salt solution (BSS). A standard phacoemulsification technique was performed thereafter through a clear corneal temporal 2.8 mm incision; consisted of anterior continuous curvilinear capsulorrhexis (CCC), nucleus emulsification, and cortex irrigation-aspiration and implantation of an acrylic IOL implant. Postoperatively, topical antibiotic (Ofloxacin 0.3%) and steroid (Prednisolone acetate 1%) medications were administered four times daily for 2 weeks and the dose was steadily reduced thereafter. As these medications were used temporarily only, we assumed that they were not a confounding factor contributing to the postoperative ocular surface changes; moreover topical steroids have been recently considered a main line in treating meibomian gland dysfunction and dry eye syndrome.

Tear film stability was assessed by the tear break-up time test which measures the interval between instillation of a sterile fluorescein strip moistened with saline applied to the inferior cul-de-sac and appearance of the first dry spots on the cornea; examination done using the cobalt-blue filter of the slit-lamp counting time in seconds needed for the first break of the precorneal tear film in a steady maintained gaze.

Tear volume was determined using basic Schirmer’s test in which sterile graded Schirmer’s paper strips placed in the lower fornix while the patient is asked to close his eyes for 5 min after which the paper is removed and amount of wetting is measured.

We quantitatively assessed the tear film stability and production by TBUT and basic Schirmer’s test respectively both preoperatively; at 1, 4 and 12 weeks postoperatively.

### Statistical analysis

The Statistical Package of Social Sciences (IBM SPSS Statistics for Windows, Version 25.0. Armonk, NY: IBM Corp.) was adopted for tabulation and analysis the obtained data. Quantitative data were presented as mean ± standard deviation while qualitative data were expressed as number (n) and percentage (%). Kolmogorov- Smirnov for normality test was used to differentiate between parametric data and non-parametric data.

Paired Samples Student T-test was used to compare preoperative and postoperative values. Repeated measures analysis of variance (ANOVA) was used to evaluate the changes over time. A *P* value less than 0.05 was considered statistically significant.

The sample size in the study provided 95.6% statistical power at the 5% level to detect a 1-s difference in tear break-up time (TBUT), when the standard deviation (SD) of the mean difference was 1 s. Results.

We have compared 21 eyes of 15 patients who had vertical LRIs (Group-A) to age-matched 21 eyes of 13 patients who had horizontal LRIs (Group-B) simultaneously with phacoemulsification; we found statistically significant reduction in the tear film stability measured by TBUT preoperatively; at 1, 4 and 12 weeks postoperatively (*P* = 0.001). No statistically significant differences regarding Schirmer’s test except in the first postoperative week in Group-B (*P* = 0.04).

In group-A who had vertically-oriented LRIs; the preoperative TBUT was 7.41 ± 2.48 s; 4.94 ± 2.36, 4.82 ± 1.94 and 5.59 ± 1.77 s, 1, 4 and 12 weeks postoperatively, (ANOVA, *p* = 0.01). the preoperative Schirmer’s test value was 14.71 ± 8.86 s; 12.88 ± 8.05, 13.53 ± 8.97 and 12.35 ± 8.20 s, 1, 4 and 12 weeks postoperatively, (ANOVA, *p* = 0.88).

In group-B who had horizontally-oriented LRIs; the preoperative TBUT was 8.81 ± 5.59 s; 6.38 ± 3.79, 7.19 ± 6.45 and 4.50 ± 3.48 s, 1, 4 and 12 weeks postoperatively, (ANOVA, *p* = 0.04). the preoperative Schirmer’s test value was 16.13 ± 9.32 s; 10.56 ± 5.16, 10.63 ± 4.77 and 9.75 ± 6.57 s, 1, 4 and 12 weeks postoperatively, (ANOVA, *p* = 0.05).

Table 2 shows the preoperative TBUT and tear volume values compared to the postoperative values as well as analysis of the time-course changes compared between both study groups.

**Table 2:** Postoperative tear film time-course changes compared to preoperative values between study groups

	Preoperative	1-Week	4-Weeks	12-Weeks	Overall P-value	Preop. Vs. 1 WK	Preop. Vs. 4 WKs	Preop. Vs. 12 WKs
**Group-A**								
**TBUT (Sec)**	7.41 ± 2.48	4.94 ± 2.36	4.82 ± 1.94	5.59 ± 1.77	0.01	0.01	0.003	0.001
**BST (mm)**	14.71 ± 8.86	12.88 ± 8.05	13.53 ± 8.97	12.35 ± 8.20	0.88	0.39	0.62	0.16
**Group-B**								
**TBUT (Sec)**	8.81 ± 5.59	6.38 ± 3.79	7.19 ± 6.45	4.50 ± 3.48	0.04	< 0.001	0.03	0.001
**BST (mm)**	16.13 ± 9.32	10.56 ± 5.16	10.63 ± 4.77	9.75 ± 6.57	0.05	0.04	0.18	0.05

*TBUT* Tear Break-up time test, *Sec* second, *BST* Basic Schirmer’s test, *mm* millimeters

To analyze the course of changes over time, we used the multiple comparison test which was found to be significant for TBUT in both groups A and B (*P* = 0.01 and 0.04 respectively) but not for tear volume production (*P* = 0.88 and 0.05 respectively).

## Discussion

Dry eye symptoms which are commonly encountered after all types of corneal refractive procedures could disturb patients’ optimal visual function and hence performing their daily life activities; such morbidities increase proportionately with the severity of symptoms [15]. It was hypothesized that the most important factor in the pathophysiology of corneal refractive surgery-induced dry eye syndrome is the transection of corneal nerves that occurs during all these incisional procedures [16].

Park et al. have studied changes in ocular surface parameters, meibomian gland function and tear inflammatory mediators following phacoemulsification; they reported worsened ocular dryness symptoms but with gradual recovery of TBUT and corneal sensitivity threshold at 1 and 2 months postoperatively. One of the 2 study groups they included had evident dry eye before surgery which was one of the exclusion criteria for the current study [17].

Oh et al. found no difference between the mean preoperative and postoperative Schirmer’s test values following phacoemulsification; however, the TBUT values were significantly decreased at 1 day postoperatively but recovered to the preoperative level after 1 month. Interestingly; they found a reduction in the mean goblet cell density which was correlated with operative time and had not recovered at 3 months postoperatively [18].

Liu et al. also reported initial significant reduction of TBUT and increased Schirmer’s test values at 1 and 2 days postoperatively with later recovery to the preoperative values [19].

To the best of our knowledge we could not find previously published reports in literature studied the ocular surface quality profile after scleral tunnel or small-incision cataract surgery. We postulate that it would not have that much effect on ocular surface profile comparable to phacoemulsification surgery which may be explained by location of the main incision being through the sclera and corneal stroma rather than incising through the limbus or clear cornea which provide the entry ports for the nerve endings.

Even with considering the latest technology of femtosecond laser-assisted cataract surgery (FLACS); a study conducted by Ju et al. reported that dry eye still could develop immediately after that procedure with a peak severity on day 7 postoperatively, most signs could return to basic preoperative levels within 3 months after surgery. They measured the tear film stability using OCULUS Keratograph which was not used in the current study [20].

In a similar way; ocular surface changes after corneal laser-assisted refractive procedures have been found to be due to cutting of corneal nerves during refractive surgeries that subsequently result in suppression of the aqueous component secretion from the lacrimal gland, mucin expression on the corneal epithelial surface, and frequent blinking, previously mentioned cascade occurs because these homeostasis-maintaining mechanisms are driven by a neuronal feedback loop that is mediated by corneal sensitivity [13, 21].

Introducing the confocal microscopy technology has helped better understanding of the dry eye pathophysiology after such incisional surgeries like LRIs as it has been proved that regeneration of the intrastromal corneal nerves usually occur within 3 to 6 months which occurs in concurrence with the recovery of corneal sensitivity and restoration of the ocular surface basic preoperative levels [22].

In a previous study, we reported reduced quality of the ocular surface profile in terms of reduced tear film BUT and tear volume production after simultaneous cataract surgery with LRIs without largely affecting the corneal sensation; however, we did not consider for selective changes according to different location of the LRIs [11].

Limitations of the current study are the relatively small sample size, the uncontrolled non-randomized design, the relatively short follow-up time and the lack of a control group that consist of patients undergoing cataract surgery without LRIs. The aforementioned limitations raised the need for future longitudinal controlled cohort studies with larger sample size and a longer follow-up time is highly recommended.

## Conclusions

In conclusion, the current study indicated that simultaneous LRIs with cataract surgery could result in dry eye symptoms and reduced tear film stability which probably would be transient during the early postoperative period and then recover to around the basic preoperative levels soon; those changes differ; however slightly; according to the LRIs orientation. Adequate preoperative assessment of the ocular surface quality parameters should be considered to optimize the postoperative outcome so not to compromise the patient’s life style.

## Data Availability

The datasets analyzed during the current study are available from the corresponding author on reasonable request.

## Abbreviations

LRIs: Limbal relaxing incisions
TBUT: Tear break-up time
BST: Basic Schirmer’s test
IOL: Intraocular lens
BSS: Balanced salt solution
CCC: Continuous curvilinear capsulorhexis
ANOVA: Analysis of variance
SD: Standard deviation
n: Number
%: Percentage
sec: Second
FLACS: Femtosecond laser-assisted cataract surgery

## References

[1] Ipek T, Hanga MP, Hartwig A, Wolffsohn J, O'Donnell C (2018). Dry eye following cataract surgery: the effect of light exposure using an in-vitro model. Cont Lens Anterior Eye.

[2] Steinert RF (2004). Cataract surgery: technique, complication and management.

[3] Bobrow JC, Blecher MH, Glasser DB, Mitchell KB, Rosenberg LF, Isbey EK. In: Surgery for cataract. Lens and cataract. 2008-2009. Section 11. Singapore; American Academy of Ophthalmology (AAO); 2008:160.

[4] Hoffer KJ (1980). Biometry of 7,500 cataractous eyes. Am J Ophthalmol.

[5] Muller-Jensen K, Fischer P, Siepe U (1999). Limbal relaxing incisions to correct astigmatism in clear corneal cataract surgery. J Refract Surg.

[6] Kaufmann C, Peter J, Ooi K, Phipps S, Cooper P, Goggin M (2005). Limbal relaxing incisions versus on-axis incisions to reduce corneal astigmatism at the time of cataract surgery; the queen Elizabeth astigmatism study group. J Cataract Refract Surg.

[7] Arraes JC, Cunha F, Azevedo Arraes T, Cavalvanti R, Ventura M (2006). Limbal relaxing incisions during cataract surgery: one-year follow-up. Arq Bras Oftalmol.

[8] Nichamin LD (2006). Astigmatism control. Ophthalmol Clin N Am.

[9] Zare MH, Tehrani MH, Gohari M (2010). Management of corneal astigmatism by limbal relaxing incisions during cataract surgery. Iran J Ophthalmol.

[10] Brint SF (1994). Refractive cataract surgery. Int Ophthalmol Clin.

[11] Ali M, Abdelhalim A (2017). Ocular surface changes after simultaneous cataract surgery and limbal relaxing incisions. J Egypt Ophthalmol Soc.

[12] Muller LJ, Vrensen GF, Pels L, Cardozo BN, Willekens B (1997). Architecture of human corneal nerves. Invest Ophthalmol Vis Sci.

[13] Donnenfeld ED, Solomon K, Perry HD (2003). The effect of hinge position on corneal sensation and dry eye after LASIK. Ophthalmology..

[14] Lee KW, Joo CK (2003). Clinical results of laser in situ keratomileusis with superior and nasal hinges. J Cataract Refract Surg.

[15] Mertzanis P, Abetz L, Rajagopalan K (2005). The relative burden of dry eye in patients’ lives: comparisons to a U.S. normative sample. Invest Ophthalmol Vis Sci.

[16] Wilson SE, Ambrosio R (2001). Laser in situ keratomileusis-induced neurotrophic epitheliopathy. Am J Ophthalmol.

[17] Park Y, Hwang HB, Kim HS. Observation of influence of cataract surgery on the ocular surface. PLoS One. 2016;11(10):e0152460. 10.1371/journal.pone.0152460 PMC5047633 27695117

[18] Oh T, Jung Y, Chang D, Kim J, Kim H (2012). Changes in the tear film and ocular surface after cataract surgery. Jpn J Ophthalmol.

[19] Liu Z, Luo L, Zhang Z (2002). Tear film changes after phacoemulsification. Zhonghua Yan Ke Za Zhi.

[20] Ju RH, Chen Y, Chen HS (2019). Changes in ocular surface status and dry eye symptoms following femtosecond laser-assisted cataract surgery. Int J Ophthalmol.

[21] Konomi K, Chen LL, Tarko RS (2008). Preoperative characteristics and a potential mechanism of chronic dry eye after LASIK. Invest Ophthalmol Vis Sci.

[22] Lee B, Mclaren J, Eric J, Hodge D, Bourne W (2002). Re-innervation in the cornea after LASIK. Invest Ophthalmol Vis Sci.

